# Weft-Knitted Spacer Fabric for Highly Stretchable–Compressible Strain Sensor, Supercapacitor, and Joule Heater

**DOI:** 10.3390/nano12203684

**Published:** 2022-10-20

**Authors:** Lu Dou, Zhen Zeng, Deshan Cheng, Shengyu Li, Wei Ke, Guangming Cai

**Affiliations:** State Key Laboratory of New Textile Materials and Advanced Processing Technologies, School of Textile Science and Engineering, Wuhan Textile University, Wuhan 430200, China

**Keywords:** wearable electronic, weft-knitted spacer fabric, strain sensor, carbon nanotubes

## Abstract

The development of wearable electronic devices has greatly stimulated the research interest of textile-based strain sensors, which can effectively combine functionality with wearability. In this work, the fabrication of highly stretchable and compressible strain sensors from weft-knitted spacer fabric was reported. Carbon nanotubes and polypyrrole were deposited on the surface of fabric via an in situ polymerization approach to reduce the electrical resistance. The as-fabricated WSP-CNT-PPy strain sensor exhibits high electrical conductivity and stable strain-sensing performance under different stretching deformations. The WSP-CNT-PPy strain sensor can be stretched up to 450% and compressed to 60% with a pressure of less than 50 KPa, which can be attributed to the unique loop and interval filament structures. The distinguishing response efficiency of WSP-CNT-PPy can effectively detect faint and strenuous body movements. In addition, the electrochemical behavior of WSP-CNT-PPy was also characterized to study the comprehensive properties. The electro-heating performance was also evaluated for feasible Joule heater applications. This work demonstrates the practicability of WSP-CNT-PPy strain sensor fabric for real-time monitoring in promising wearable garments.

## 1. Introduction

In the past few years, wearable electronic devices have gradually emerged as a field of interest, which has received tremendous attention with respectable progress [1,2,3,4]. Among various subassemblies, the wearable strain sensor is the foundation for fabricating wearable garments and it is effective for converting external mechanical force into recognizable real-time electrical signals [5,6]. Considering the excellent skin-friendly features and flexibility, electronic textiles have emerged as the next generation of wearable devices. Textile-based systems can effectively integrate the multi-functional properties of electronic devices for various applications including energy storage and conversion [7,8], healthcare monitoring [9,10,11], visualization signal displays [12,13], soft robotics [14,15], human–machine interfaces [16,17,18], human body movement detection [19,20,21], etc. Textile sensors can provide a relatively facile fabrication approach and diverse structure transformations by varying the arrangements of fibers and yarns. For human motion monitoring, textile strain sensors are highly sensitive to detect both tiny and large strain deformations, which is an advantage of the textile skeleton frame [22,23,24,25].

Generally, knitted textiles can be intrinsically extended in both weft and warp directions due to the loop constructions. In the past decades, fabrication of knitted fabric strain sensors for human body movement detection has been a popular research topic [26,27,28,29]. The structure of a weft-knitted spacer fabric consists of two outer layers connected by floats of yarn laid between two needle beds in the inner layer. Generally, weft-knitted spacer fabric exhibits good comfort qualities, and is suitable for garment applications. It has also exhibited good washability and stability due to the unique spacer structure. In our previous studies, weft-rib-knitted fabric was manufactured from spandex filament, which acted as the stretchable component in the core. Both the intrinsic elasticity of filament and loops can contribute to the highly stretchable performance of the strain sensor [30]. Ye et al. [31] reported a stretchable, washable, and rapid sensitive wool-knitted fabric sensor modified by graphene that exhibits high linearity with more than 20% elongation with moisture from 30 to 90%. Flexible knitted fabric strain sensors can also be used for identifying knee joint motion patterns, which are promising for human body health monitoring [32,33]. Furthermore, copper nanoparticles deposited on knitted fabric [5], knitted conductive gloves [34], and polypyrrole-coated knitted cellulose fabric [35] were also fabricated to construct a strain-sensing system for wearable applications. However, most of these textile-based strain sensors are designed for onefold stretchable sensing applications. In practical applications, the deformation is generally complicated. With the elongation of a strain sensor, the thickness will also be decreased, which is closely related to the compression property. In addition, a compressible strain sensor is favorable for greater human motion detection, but it is difficult to fabricate a compressible fabric sensor. Similar to stretchability, compressibility is also an essential characteristic to realize the accurate detection of wearable electronics.

According to the reported literature, compressible strain sensors were conventionally made of elastic polymer materials. An aerogel sensor consisting of aramid nanofibers was successfully fabricated, which can be used for wearable electronics in detecting pressure with smart shoes and insoles [36]. Pan et al. [37] prepared composite hydrogels with improved mechanical properties that can be stretched to a strain of more than ten times and compressed to a strain of 80%. In addition, a compressible MXene aerogel-based piezoresistive pressure sensor [38], compressible and conductive self-healing hydrogels strain sensor [39], and compressible integrated sponge supercapacitor sensor [40] was also fabricated for wearable applications in different fields. Jiang et al. [41] reported the preparation of hierarchical three-dimensional graphene fiber assemblies, where the fiber-to-fiber interfacial region produces a highly sensitive contact resistance, thus leading to a higher sensitivity. However, this porous structure is difficult to apply directly for garments and the wearability should be improved. Therefore, it is essential to develop textile strain sensors for wearable e-textile applications. Spacer-knitted fabrics are usually obtained by the combination of two fabric layers, while weft-knitted spacer fabrics (WSP) are made by knitting two single-jersey fabrics, and interval filaments are stitched to connect the double-faced layers [42]. Considering the excellent compressibility, weft-knitted spacer fabric has been considered as an alternative choice for compressible strain sensors. Polypyrrole (Ppy) is one of the most extensively used conducting polymers in design and fabrication of various types of electro-chemical sensors and biosensors. It can be easily generated and uniformly deposited on the conducting surfaces of various matrix materials. Furthermore, it has also exhibited the combination of high electrical conductivity and polymeric properties including good flexibility, low density, and environmental stability. In the present work, it has demonstrated a highly stretchable and compressible strain sensor to detect both elongation and pressure signals. The strain sensor was based on WSP, and polypyrrole was in situ deposited on the surface to obtain high electrical conductivity and electrochemical activity. Furthermore, it has also exhibited robust electro-heating effects for potential Joule heater applications.

## 2. Experimental

### 2.1. Materials

Weft-knitted spacer fabric in this study was purchased from Alibaba, Co., Ltd., China. The superficial layer of weft-knitted spacer fabric is polyamide filament with the specifications of 240D/36F, and the interval yarn is polyester filament with a diameter of 60 μm. The thickness of the weft-knitted spacer fabric is 4.0 mm, and the surface area weight is 320 g/m^2^. Single-walled carbon nanotubes (0.15 wt %, external diameter of 1–2 nm, length of 5–30 μm, purity of higher than 95%) obtained from Nanjing XFNANO Materials Tech Co., Ltd., China. Pyrrole and Cetyltrimethylammonium bromide (CTAB) exhibited the purity of chemical grade. Sodium dodecylbenzene sulfonate (SDBS), FeCl_3_·6H_2_O, phosphoric acid, and anhydrous ethanol were obtained from Pharmaceutical Group Chemical Reagents Co., Ltd., Shanghai, China.

### 2.2. Preparation of Weft-Knitted Spacer Fabric Sensor

**Figure 1:**
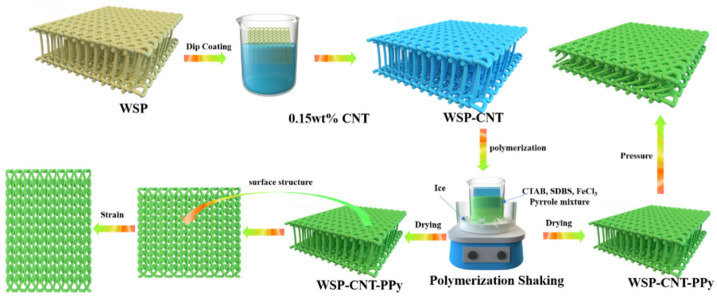
Diagram of the fabrication process of the WSP-CNT-PPy strain sensor.

The WSP was firstly pretreated with an acetone solution for 5 h, dried in natural conditions, then repeatedly soaked in 0.15 wt % single-wall carbon nanotube solutions to obtain stable electrical resistance. After a 12 h period of drying at 70 ℃, the treated sample was denoted as the carbon nanotube weft spacer fabric (WSP-CNT). An amount of 6 mL of pyrrole was poured into 60 mL of deionized water with 0.208 g CTAB and 0.216 g SDBS dispersant under magnetic stirring conditions. An amount of 1 cm × 2 cm WSP-CNT was placed into pyrrole solution for a 2 h ice bath shock. An amount of 0.5 mol/L ferric chloride solution of 60 mL was slowly dropped into the pyrrole solution for an in situ polymerization reaction. The sample was washed with anhydrous ethanol and deionized water three times, and then dried at 70 °C for 12 h to prepare the polypyrrole-deposited WSP-CNT (WSP-CNT-PPy), as shown in Figure 1.

### 2.3. Measurements and Characterization

The microstructures and surface morphologies of the prepared samples were characterized by scanning electron microscopy (SEM, JSM5600LV, JEOL, Tokyo, Japan). The tensile and compression properties of the samples were measured by Instron equipment. The electrical resistance was measured by an electrochemical workstation of CHI604E, and the corresponding resistance variation by digital multimeter. Ω/cm is the most widely used unit to characterize the surface electrical resistance, therefore, the unit of Ω/cm was used to measure the resistance of the strain sensors. The cyclic voltammetry (CV), electrochemical impedance spectroscopy (EIS), and galvanostatic charge-discharge (GCD) were carried out. The thermal imaging and photo-induced heating performance of the WSP-CNT-PPy strain sensor was characterized by an infrared thermal camera (FLIR ONE Pro), and both the surface temperature and thermal images were recorded. All experiments were performed under the standard temperature of 25 °C and humidity of 65%.

## 3. Results

### 3.1. Surface Morphologies, Microstructures, and Mechanical Properties

**Figure 2:**
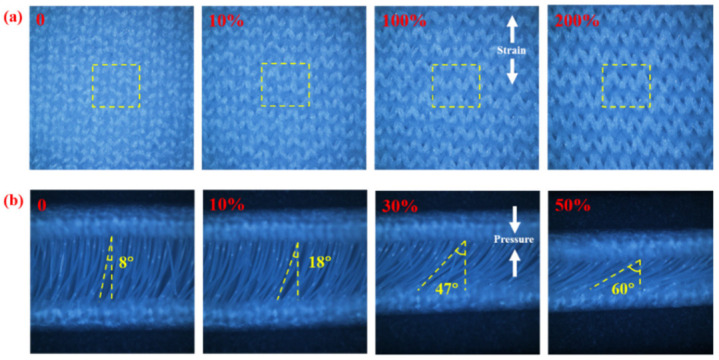
(**a**) Optical images of the WSP strain sensor under different elongation conditions and (**b**) inclination angle of interval filament under different compression conditions.

The optical images of the WSP strain sensor under both unstretched and stretched in weft direction conditions are shown in Figure 2a. The surface morphologies of the strain sensor with increasing strain levels of 0%, 10%, 100%, and 200% are observed. With the elongation of the applied strain, the series of loops were stretched in the direction of loading. The inclination angle of the interval filament gradually increased under applied pressure and the compression was 0%, 10%, 30%, and 50%, respectively. In Figure 2b, the measured angle is approximate 8°, 18°, 47°, and 60°, which indicates the excellent compressible properties of the WSP-CNT-PPy strain sensor.

**Figure 3:**
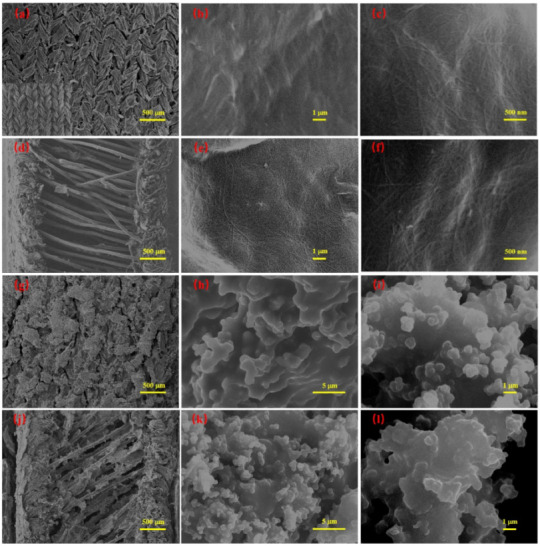
SEM images of pristine weft-knitted spacer fabric (**a**–**c**) surface and cross section (**d**–**f**) at different magnifications and SEM images of the WSP-CNT-PPy strain sensor (**g**–**i**) surface and cross section (**j**–**l**) at different magnifications.

The microscopic morphologies of the WSP-CNT and WSP-CNT-PPy strain sensors were characterized by SEM, as shown in Figure 3. In Figure 3a–f, the surface-knitted structure and interval filaments of the pristine weft-knitted spacer fabric can be clearly seen from the SEM images. The surface microstructures of the WSP-CNT-PPy strain sensor at different magnifications are shown in Figure 3g–i. The high-magnification image of the WSP-CNT-PPy strain sensor indicates the uniform distribution of the CNT-PPy composite layer on the fabric surface. The grainy microstructure of the fabric is beneficial to improve the deposition of the strain-sensing layer in the following fabrication step. In Figure 3j–l, interval filaments of the WSP-CNT-PPy strain sensor were also fully covered with the CNT-PPy composite layer. The layer can be strongly adhered to the filament and it plays the role of a conductive layer during the compression process. The amplified images of the CNT-PPy are shown in Figure 3k,l, which shows a uniform dispersion of CNT-PPy nanoparticles, therefore, increasing the conductivity and strain-sensing properties.

**Figure 4:**
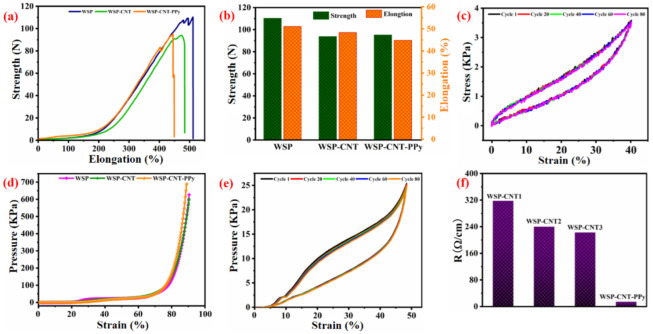
(**a**,**b**) Mechanical strength of WSP, WSP-CNT, and WSP-CNT-PPy; (**c**) cyclic stress–strain curves; (**d**) pressure under different strains of WSP, WSP-CNT, and WSP-CNT-PPy; (**e**) cyclic pressure–strain curves; and (**f**) electrical resistance of WSP-CNT dipped into 0.15 wt % CNT solutions for 1 to 3 times (denoted as WSP-CNT1, WSP-CNT2, and WSP-CNT3, respectively) and WSP-CNT-PPy.

Excellent mechanical performance has been considered an important feature in the engineering of wearable applications. The mechanical properties of WSP, WSP-CNT, and WSP-CNT-PPy were studied. In Figure 4a, it can be seen that the elongation is higher than 450% in the weft direction under a force higher than 90 N, where the cross-sectional length is 2 cm. The obtained strength and elongation rate are shown in Figure 4b. Compared with the pristine WSP, the mechanical properties of the WSP-CNT-PPy strain sensor were slightly decreased, with the tensile strength of 92 N, a cross-sectional length of 2 cm, and elongation at a break of 450%. This can be attributed to the surface structure being damaged by the nanoparticles due to the interfacial effects. The WSP-CNT-PPy strain sensor is elongated up to 450% with the unique loop structure of the weft-knitted spacer fabric. The cyclic stress–strain curves are shown in Figure 4c, where the strain ranges from 0 to 40% with the stress from 0 to 3.5 KPa. The result indicates the robust durability of the WSP-CNT-PPy strain sensor under 80 cycles of press and release experiment. It can be stated that all three samples can bear the forced pressure of higher than 600 KPa with 90% strain, as shown in Figure 4d. With a pressure of 25 KPa, the WSP-CNT-PPy strain sensor exhibited good stability during 80 cycles of pressure-releasing treatment. It is feasible to achieve sensing efficiency with the compressible strain sensor in practical applications. Figure 4f shows that the fabricated WSP-CNT-PPy strain sensor exhibited decreased electrical resistance (from 318 Ω/cm, 240 Ω/cm, and 223 Ω/cm to 14.18 Ω/cm), thus meeting the requirements of wearable electronics applications.

### 3.2. Stretchable Strain Sensing and Wearable Applications

**Figure 5:**
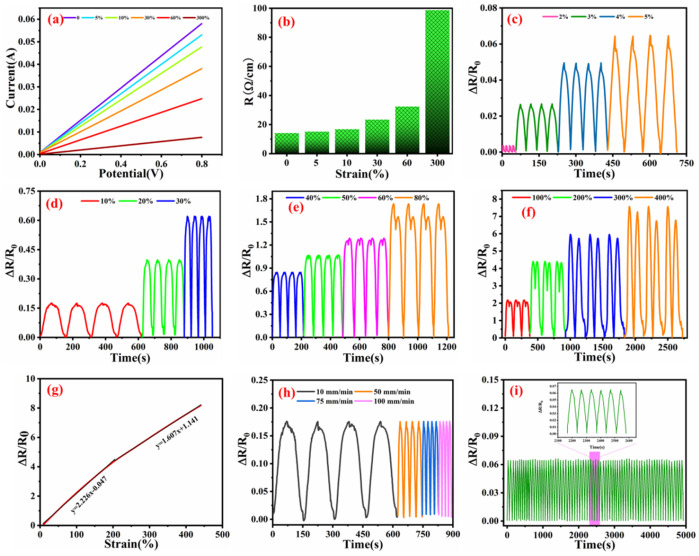
(**a**) I–V curves of elongation; (**b**) resistance of different strain conditions; (**c**–**f**) ΔR/R0 vs. cyclic strains of 2%, 3%, 4%, 5%, 10%, 20%, 30%, 40%, 50%, 60%, 80%, 100%, 200%, 300%, and 400%; (**g**) function of relative resistance charge (ΔR/R_0_); (**h**) ΔR/R_0_ with 10% cyclic strain at various loading conditions; and (**i**) stability of the WSP-CNT-PPy strain sensor under a cyclic strain of 5%.

The I–V curves are shown in Figure 5a. With the increasing elongation process, the slope line was gradually decreased, and a representative linear behavior was observed. In Figure 5b, the resistance value was tardily elevated with the increasing elongation process. As the stretching progressed, both knitted fabric and yarn became thinner, thus the electrical resistance was also improved. The good deformation of the WSP-CNT-PPy with unique knitting loop structures is beneficial to applications. It can also ensure the high strain sensitivity and durability of the WSP-CNT-PPy fabric strain sensor. The detailed spectrum of strain sensor relative resistance variation under cyclic stretching–releasing strain is shown in Figure 5c–f. The detailed curves under the cyclic strains of 2%, 3%, 4%, 5%, 10%, 20%, 30%, 40%, 50%, 60%, 80%, 100%, 200%, 300%, and 400% are measured and presented. This indicates that the ΔR/R_0_ gradually increases with the increase in strain elongation. However, the culmination of the ΔR/R_0_ curve sags in the middle and it becomes increasingly obvious with higher tensile strain. This phenomenon can be attributed to the inhomogeneity of the knitting loops of weft-knitted spacer fabric under strain conditions. These measured results are identical with the measured values in Figure 5g. The electrical resistance variation (ΔR/R_0_) of the WSP-CNT-PPy is shown in Figure 5g. The WSP-CNT-PPy strain sensor exhibits a wide strain sensing range and it can be observed from measured and fitted curves that there are two stages of ΔR/R_0_ ranging from 0 to 450%. The stages of 0–200% and 200–450% are related to a gauge factor (GF) of 2.226 and 1.607, respectively. The variation in the ΔR/R_0_ can be attributed to the distortion of the knitted structure due to loading force. Compared with reported studies, this indicates that the WSP-CNT-PPy strain sensor exhibits a large sensing deformation. For instance, Souri et al. [43] reported a stretchable strain sensor based on conductive cotton and wool fabric, where the strain range is 0–150%. The conductive polyester fabric strain sensor fabricated from a weft-knitted structure exhibited a high stretchability up to 130% with the loading of increased strain [44]. The slow-motion deformation of loops is related to the variation in the interaction between different materials. As the stretching process continues, the ΔR/R_0_ also gradually increases.

In detail, the strain sensor fabric was initially elongated, and the knitted loops were divided step-by-step. The forced stretching led to the crack of conductive materials and abruption of the deposited nanoparticles. Thus, the sample performance mainly included two steps of 0–200% and 200–450%. Both high sensitivity and wide strain sensing range are important parameters in detecting human motions. The sensitivity of the WSP-CNT-PPy strain sensor to various stimuli conditions was evaluated with the periodic pressure of 10%, and the resulting loading speed was 10 mm/min, 50 mm/min, 75 mm/min, and 100 mm/min, respectively, as shown in Figure 5h. It has been suggested that the sensor shows stable ΔR/R_0_ data with periodic traction and relaxation of different loaded conditions. The result indicates that the WSP-CNT-PPy exhibits a reliable response under various external stimuli, thus ensuring reliability in practical applications.

In addition, the cyclic stability of the WSP-CNT-PPy was further measured in Figure 5i. The performance of the sensor under 5% periodic compressive stress at the loading speed of 10 mm/min was recorded. Furthermore, the ΔR/R_0_ value exhibits a constant increase and decrease in every experiment, which demonstrates the robust reproducibility of the strain sensor.

**Figure 6:**
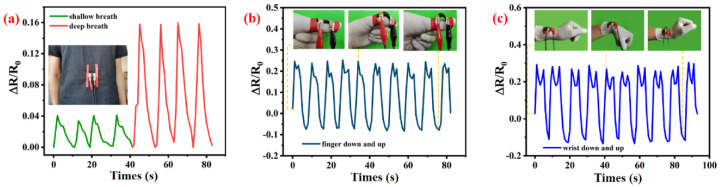
Detection of various human body movements including: (**a**) shallow-deep breath, (**b**) finger up-down, and (**c**) wrist up-down.

To further investigate the promising wearable applications of the WSP-CNT-PPy strain sensor, a strain-sensing method to monitor various human movements was established. The WSP-CNT-PPy-based strain-sensing device exhibited high sensitivity in recognition of tiny motions. In Figure 6a, the WSP-CNT-PPy strain-sensing equipment was inserted into the cloth of the abdomen to monitor the response to both shallow and deep breath. The result indicates that the respiratory state can be accurately reflected by the magnitude of the recorded spectrum. The magnitude of ΔR/R_0_ within 0–0.04 corresponds to shallow breath, while the ΔR/R_0_ within 0–0.16 corresponds to deep breath. The actual time variation of the ΔR/R_0_ data with human breath indicates the quick induction and good sensitivity of the WSP-CNT-PPy. Figure 6b,c correspond to the spectra of ΔR/R_0_ responses for finger down and wrist up–down motion, respectively. The results indicate that the spectra are distinctive for different movements. Moreover, the spectra have also exhibited robust repeatability in the continuous up–down process. The ΔR/R_0_ data cyclically varied corresponding to the movement and resulted in a highly periodical spectra. The tiny movements of the breath, finger, and wrist were reflected by the observed curve of ΔR/R_0_, which indicates the shrinkage of muscles or skeleton. These measured results indicate that the strain-sensing system is effective to detect various human motions.

### 3.3. Compressive Strain Sensing and Wearable Applications

**Figure 7:**
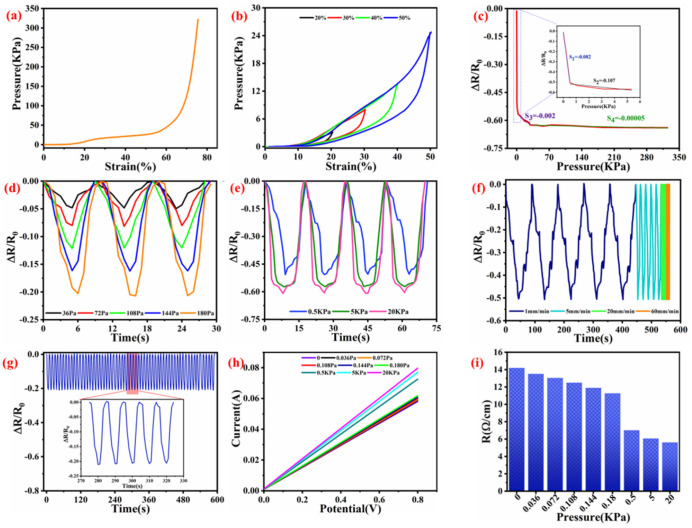
(**a**) Pressure-strain curve; (**b**) cyclic pressure-strain curve under the compression strain of 20%, 30%, 40%, and 50%; (**c**) relative resistance change (ΔR/R0) as a function of pressure; (**d**,**e**) ΔR/R_0_ under a cyclic pressure of 36 Pa, 72 Pa, 108 Pa, and 180 Pa, and 0.5 KPa, 5 KPa, and 20 KPa; (**f**) ΔR/R0 change with the cyclic pressure of 0.5 KPa under 1 mm/min, 5 mm/min, 20 mm/min, and 60 mm/min loading speed; (**g**) durability of WSP-CNT-PPy under 180 KPa pressure; (**h**) I-V curves with various pressure; and (**i**) resistance with different pressure.

The robust compression capability of fabric has been considered as the foundation to fabricate a compressive strain sensor. Both high compression sensitivity and wide strain range are important parameters for enhancing the sensing efficiency. With the increase in forced pressure, the change of strain is shown in Figure 7a. It can be seen that the WSP-CNT-PPy is easy to be compressed to 60% with a pressure of less than 50 KPa. For larger deformation, the required pressure is quickly increased. The compression recovery curves with different pressure were also measured, where the strain value is 20%, 30%, 40%, and 50% as shown in Figure 7b. The results indicate the stable response performance of WSP-CNT-PPy, which can meet practical wearable applications. Figure 7c shows the electrical resistance variation (∆R/R_0_) of the WSP-CNT-PPy strain sensor when subjected to forced pressure. It can be seen that the curve was divided into four stages: S1 = −0.082, S2 = −0.107, S3 = −0.002, and S4 = −0.00004, respectively. The ΔR/R_0_ shows a linear decrease against the increasing compressive pressure in different ranges. To investigate the properties in practical applications, the ΔR/R0 responses to repeated compressing and releasing cycles are illustrated in Figure 7d,e. The results indicate that a slight stress of 36 Pa can be effectively detected by the WSP-CNT-PPy strain sensor and stable resistance responses are observed under different compressive pressure, thus it can be used as a detectable pressure sensor.

The compress–release curves with pressures of 36 Pa, 72 Pa, 144 Pa, and 180 Pa are shown in Figure 7d. These resistance responses of WSP-CNT-PPy to small compressive pressure show potential applications in detecting weak motions, such as facial expression and language communication. As the compressive stress increases to 0.5 KPa, 5 KPa, and 20 KPa, the intensity of the signal becomes higher and the stability is also improved. From the large repeated strains, a series of resistance responses can also be obtained from Figure 7e. The result indicates that a higher strain causes a higher electrical resistance response intensity, and it is quite stable under identical pressure conditions. These distinguishing response signals under different pressures make the WSP-CNT-PPy capable of detecting strenuous exercise and accurately reflecting large-scale human movements. With the pressure of 5KPa, the ΔR/R_0_ spectrum of WSP-CNT-PPy is recorded, where the loading speed is 1 mm/min, 5 mm/min, 20 mm/min, and 60 mm/min, respectively.

In addition, the cyclic compression stability of the WSP-CNT-PPy was recorded in Figure 7g. The performance of the sensor with 180 Pa stress at the loading speed of 5 mm/min was tested. It indicates that the ΔR/R_0_ signal exhibits good reproducibility for at least 10 min. Furthermore, the current–voltage (I–V) curve of WSP-CNT-PPy under different compressive pressure has been studied, as shown in Figure 7h. It can be seen that WSP-CNT-PPy exhibits good linear I–V characteristics under different compressive stress values of 0 to 20 KPa. The slope was gradually increased with the increasing compressive stress, which corresponds to the decrease in electrical resistance, as shown in Figure 7i. The ultra-wide compressive deformation of the WSP-CNT-PPy sensor is due to the interval filaments of three-dimensional knitted spacer fabric. Therefore, the WSP-CNT-PPy fabric with high-strain sensitivity and durability can be used as a high compression strain sensor in wearable garments.

**Figure 8:**
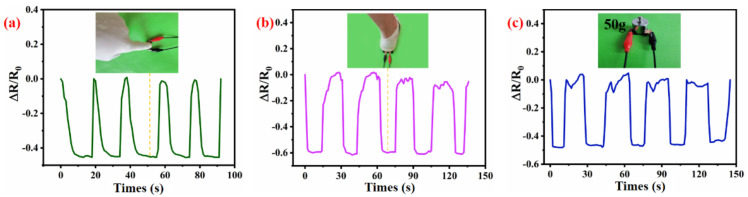
Detection of various compression movements including (**a**) finger press, (**b**) foot press, and (**c**) counterweight press.

The integrated strain-sensing equipment was taken to evaluate the compression sensing efficiency of WSP-CNT-PPy. The result indicates that the WSP-CNT-PPy exhibited good sensitivity to recognize different compression behavior. In Figure 8a, the WSP-CNT-PPy strain sensor was used to measure the finger compression and response behavior. It can be seen that the respiratory state was accurately monitored by the magnitude of the spectrum. The cyclic variation of ΔR/R_0_ corresponds to 0 to negative 0.4 with the behavior of compressing and releasing. The ΔR/R_0_ value with the pressing process indicates good sensitivity properties of the WSP-CNT-PPy compression sensor. Figure 8b is the measured curve of ΔR/R_0_ related to foot down and up, where the cyclic variation of ΔR/R_0_ corresponds to zero to negative 0.6. It can be stated that the compressive stress is higher than the finger. Figure 8c is the measured spectrum corresponding to a 50 g counterweight. These distinctive spectrums have also exhibited good repeatability in the continuous down–up compression process. The ΔR/R_0_ curve was cyclically changed due to the compression behavior, thus to obtain a highly periodical spectrum. This indicates that the WSP-CNT-PPy sensor is effective in detecting compression behaviors in different compressive strain-sensing applications.

### 3.4. Electrochemical Performance of WSP-CNT-PPy

**Figure 9:**
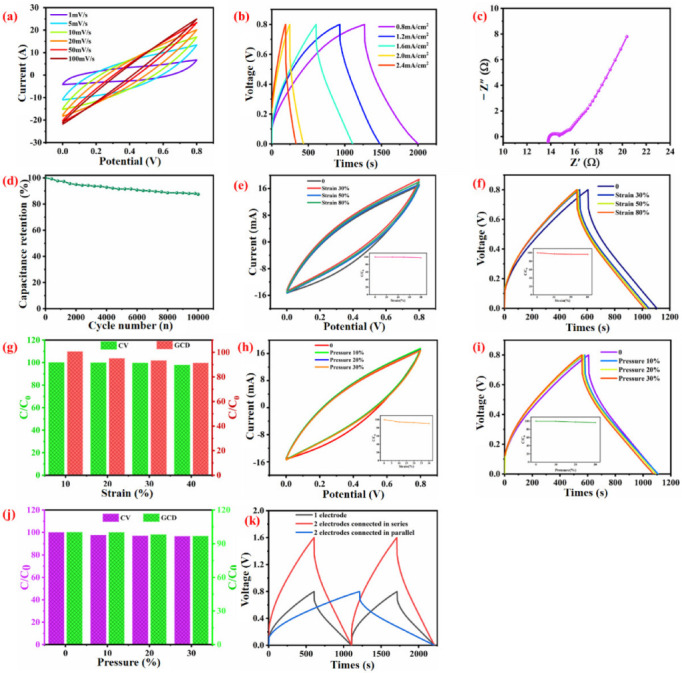
(**a**) I-V curves of WSP-CNT-PPy under various rates; (**b**) GCD curves with different areal currents; (**c**) Nyquist plot under the amplitude of 50 mV/s; (**d**) cycle stability curve; (**e**) CV curves of various strain pressure; (**f**) GCD curves of different strain conditions; (**g**) the efficiency of CV and GCD curves is maintained with the strain ranging from 0 to 40%; (**h**) CV curves under different pressure conditions; (**i**) GCD curves of different pressure conditions; (**j**) capacitance maintain of CV and GCD curves with the pressure of 0 to 30%; and (**k**) single electrode, and electrodes connected in series and parallel.

Electrochemical sensors have been widely used in various areas due to the advantages of their simplicity and convenience. The preparation of flexible electrochemical sensors is based on fibrous materials and deformability of the material is important to install the devices for consecutive charging–discharging. The electrochemical performance of the WSP-CNT-PPy fabric was evaluated by CV, GCD, and EIS methods. In detail, WSP-CNT-PPy was measured and the I–V curves at different scanning rates are shown in Figure 9a. It can be seen that the CV curves have a good symmetrical phenomenon, which indicates excellent supercapacitor performance of WSP-CNT-PPy. According to Ccv = ∫IdVQ/(2AΔU), I denotes response current (A), v denotes scan rate (mV/s), A is the areal of material (cm^2^), and ΔU is the potential working window (V). The areal capacitance of WSP-CNT-PPy at 1 mV/s is approximately 1765.6 m F/cm^2^. The GCD data of various areal currents ranging from 0.8 to 2.4 mA/cm^2^ are presented in Figure 9b. Furthermore, the typically inverted V-shape of the data has proven the slight inside resistance. According to Formula C_gcd_ = It/(AΔU), the calculated areal special capacitance C_gcd_ is 740 mF/cm^2^ at 0.8 mA/cm^2^, with a capacitance retention of 87.4% when the current density increases seven times. The ED is 65.8 μW h/cm^2^ and the PD is 320.1 μW/cm^2^ according to the equation of ED = C_gcd_ ΔU2/7200 and PD = 3600 ED/t, at a current of 0.8 mA/cm^2^.

EIS was recorded under the frequency between 0.01 and 100 kHz of 50 mV current, as shown in the Nyquist plot of Figure 9c. The result indicates that WSP-CNT-PPy exhibits good supercapacitor behavior. The periodic curve of electrochemical properties is shown in Figure 9d, after 10,000 times experiments, the retention productivity is approximate 88%, indicating a good reversibility and long cycling stability. Figure 9e,f presents the recorded CV and GCD curves with various strain–stress up to 80%, and the curves show slight variation corresponding to various strain-sensing conditions. Furthermore, the illustrations in Figure 9e,f indicate good stability. The capacitance retention of CV and the GCD curves are presented in Figure 9g. This result suggests stable electrochemical efficiency under a strain between 0 and 40% and this is due to the special knitting loops of the weft-knitted spacer fabric. Figure 9h–k present the electrochemical properties of WSP-CNT-PPy under different pressure conditions, which are similar to strain conditions. The good electrochemical behavior is due to the unique structure of the spacer fabric and electrical conductivity of CNT-PPy. The spacer knitted fabric with CNT-PPy composites under stretching and pressure conditions maintained good electrochemical properties.

### 3.5. Electro-Heating Performance

**Figure 10:**
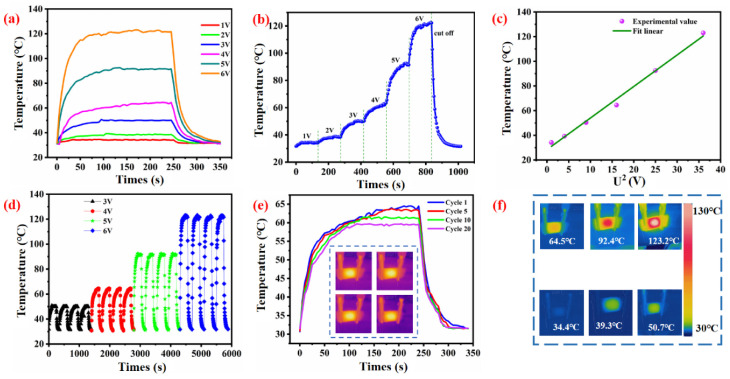
(**a**) The heating process of WSP-CNT-PPy under different applied voltage; (**b**) the increase in temperature with the increasing voltage; (**c**) experimental value and fitted linear curve between temperature and square of voltage; (**d**) heating process under cyclic voltage of 3 V, 4 V, 5 V, and 6 V; (**e**) heating process under different cycles; and (**f**) heat-imaging of the WSP-CNT-PPy fabric.

Electric heating is the process concerning the energy of electric current converted into heat, which is also referred to as Ohmic heating or Joule heating. Considering the simplicity of electrical power and relative high energy conversion efficiency, electrical heating is the most widely used method for various heating applications. The electro-heating performance of WSP-CNT-PPy was evaluated as shown in Figure 10. It can be seen that all temperature profiles exhibited a similar tendency. Once the devices are powered, the measured temperature of WSP-CNT-PPy increased simultaneously and reached a plateau in 50 s. After 250 s, the system powered off and the temperature decreased quickly, as shown in Figure 10a. The Joule heating capability of WSP-CNT-PPy was also evaluated by measuring the temperature, which was increased with time and applied voltage, as shown in Figure 10b. The generated thermal energy was gradually increased with the input voltage. According to Joule’s law, E = V^2^t/R, where E is heat produced by Joule effect, V is the applied voltage, t is time, and R is electrical resistance. Figure 10c has presented the experimental value and fitted linear curve between temperature and square voltage, and the obtained temperature can be effectively predicted when the voltage ranged from 1 V to 6 V. With the input voltage of 6 V, the WSP-CNT-PPy reached the highest temperature of 120 °C with a fast response time. The high heat generation efficiency allows for promising application of WSP-CNT-PPy in personal thermal management devices. The cyclic on–off thermal response process at an applied voltage of 3–6 V is shown in Figure 10d. The obtained temperature was gradually increased with the increasing voltage, and was maintained during the four cycles, respectively. The heating process of WSP-CNT-PPy under different cycles is shown in Figure 10e, where the applied voltage is 4 V. It indicates the excellent repeatability of WSP-CNT-PPy under reutilization conditions for long-term practical applications. The heat-imaging of the WSP-CNT-PPy fabric is shown in Figure 10f, and the voltage ranged from 1 V to 6 V. The relationship between temperature and time under various conditions of electro-heating is nearly identical, which indicates the ultra-stability and high repetitiveness of the WSP-CNT-PPy fabric. The WSP-CNT-PPy fabric could monotonically increase to the steady-state maximum temperature of 123.2 °C by 50 s under the applied 6 V electric voltage.

## 4. Conclusions

In this work, a stretchable and compressive strain sensor with good extensibility and sensitivity was prepared by employing weft-knitted spacer fabric as a matrix. Carbon nanotubes and polypyrrole were successfully deposited on the surface of the fabric through an in situ polymerization process to obtain high conductivity. The unique knitted loop structures together with interval filaments are beneficial to improve strain sensing efficiency under both tiny and high deformations. The WSP-CNT-PPy strain sensor also exhibited excellent electrochemical performance at various strain and pressure conditions. It is suggested that highly stretchable and compressive WSP-CNT-PPy can be used as a potential candidate for future wearable electronic devices. In addition, the as-fabricated WSP-CNT-PPy strain fabric also exhibited robust electrochemical and electroheating properties, and the heating/cooling process exhibits good repeatability. The present work would enable the development of next-generation carbon-nanotube-based wearable textiles for multifunctional applications.

## Data Availability

Not applicable.

## References

[1] Afroj S., Tan S., Abdelkader A.M., Novoselov K.S., Karim N. (2020). Highly conductive, scalable, and machine washable graphene-based e-textiles for multifunctional wearable electronic applications. Adv. Funct. Mater..

[2] Lee J., Llerena Zambrano B., Woo J., Yoon K., Lee T. (2020). Recent advances in 1d stretchable electrodes and devices for textile and wearable electronics: Materials, fabrications, and applications. Adv. Mater..

[3] Yang K., Isaia B., Brown L.J.E., Beeby S. (2019). E-textiles for healthy ageing. Sensors.

[4] Wang Y., Huang X., Zhang X. (2021). Ultrarobust, tough and highly stretchable self-healing materials based on cartilage-inspired noncovalent assembly nanostructure. Nat. Commun..

[5] Cheng D.S., Bai X., Pan J.J., Wu J.H., Ran J.H., Cai G.M., Wang X. (2020). In situ hydrothermal growth of cu nps on knitted fabrics through polydopamine templates for heating and sensing. Chem. Eng. J..

[6] Li X., Koh K.H., Farhan M., Lai K.W.C. (2020). An ultraflexible polyurethane yarn-based wearable strain sensor with a polydimethylsiloxane infiltrated multilayer sheath for smart textiles. Nanoscale.

[7] Tang X., Yan X. (2017). Dip-coating for fibrous materials: Mechanism, methods and applications. J. Sol-Gel Sci. Technol..

[8] Chai Z., Zhang N., Sun P., Huang Y., Zhao C., Fan H.J., Fan X., Mai W. (2016). Tailorable and wearable textile devices for solar energy harvesting and simultaneous storage. ACS Nano.

[9] Lee J., Kim D., Chun S., Song J.H., Yoo E.S., Kim J.K., Pang C. (2020). Intrinsically strain-insensitive, hyperelastic temperature-sensing fiber with compressed micro-wrinkles for integrated textronics. Adv. Mater. Technol..

[10] Hatamie A., Angizi S., Kumar S., Pandey C.M., Simchi A., Willander M., Malhotra B.D. (2020). Review—Textile based chemical and physical sensors for healthcare monitoring. J. Electrochem. Soc..

[11] Xu X., Luo M., He P., Yang J. (2020). Washable and flexible screen printed graphene electrode on textiles for wearable healthcare monitoring. J. Phys. D: Appl. Phys..

[12] Mishra R.K., Martín A., Nakagawa T., Barfidokht A., Lu X., Sempionatto J.R., Lyu K.M., Karajic A., Musameh M.M., Kyratzis I.L. (2018). Detection of vapor-phase organophosphate threats using wearable conformable integrated epidermal and textile wireless biosensor systems. Biosens. Bioelectron..

[13] An J., Ma Y., He M., Yan J., Zhang C., Li X., Shen P., Luo S., Gao Y. (2020). A wearable and highly sensitive textile-based pressure sensor with Ti3C2Tx nanosheets. Sens. Actuators A: Phys..

[14] Hassan Z., Kalaoglu F., Atalay O. (2020). Development and characterization of conductive textile (cotton) for wearable electronics and soft robotic applications. Tex. Res. J..

[15] Pyka W., Jedrzejowski M., Chudy M., Krafczyk W., Tokarczyk O., Dziezok M., Bzymek A., Bysko S., Blachowicz T., Ehrmann A. (2020). On the use of textile materials in robotics. J. Eng. Fibers Fabr..

[16] Liao X., Song W., Zhang X., Huang H., Wang Y., Zheng Y. (2018). Directly printed wearable electronic sensing textiles towards human-machine interfaces. J. Mater. Chem. C.

[17] He Q., Wu Y., Feng Z., Fan W., Lin Z., Sun C., Zhou Z., Meng K., Wu W., Yang J. (2019). An all-textile triboelectric sensor for wearable teleoperated human-machine interaction. J. Mater. Chem. A.

[18] Zhang L., Liang Y., Xiao P., Chen T., Zhang L., He J., Liang Y., Chen T., He J., Liao Y. (2019). A self-protective, reproducible textile sensor with high performance towards human-machine interactions. J. Mater. Chem. A.

[19] Liu M., Pu X., Jiang C., Liu T., Huang X., Chen L., Du C., Sun J., Hu W., Wang Z.L. (2017). Large-area all-textile pressure sensors for monitoring human motion and physiological signals. Adv. Mater..

[20] Eguchi K., Nambu M., Kamikawa T., Ueshima K., Kuroda T. (2019). Smart textile device with embedded fabric electrodes targeting periodic limb movements monitoring at home: A case report. J. Fiber Sci. Technol..

[21] Eizentals P., Katashev A., Oks A., Semjonova G. (2020). Smart shirt system for compensatory movement retraining assistance: Feasibility study. Health Technol..

[22] Zhao Z., Huang Q., Yan C., Liu Y., Zeng X., Wei X., Hu Y., Zheng Z. (2020). Machine-washable and breathable pressure sensors based on triboelectric nanogenerators enabled by textile technologies. Nano Energy.

[23] Lan L., Zhao F., Yao Y., Ping J., Ying Y. (2020). One-step and spontaneous in-situ growth of popcorn-like nanostructures on stretchable double-twisted fiber for ultra-sensitive textile pressure sensor. ACS Appl. Mater. Interfaces.

[24] Qi K., Zhou Y., Ou K., Dai Y., You X., Wang H., He J., Qin X., Wang R. (2020). Weavable and stretchable piezoresistive carbon nanotubes-embedded nanofiber sensing yarns for highly sensitive and multimodal wearable textile sensor. Carbon.

[25] Guo Q., Huang B., Lu C., Zhou T., Su G., Jia L., Zhang X. (2019). A cephalopod-inspired mechanoluminescence material with skin-like self-healing and sensing properties. Mater. Horiz..

[26] Ma P.B., Chang Y.P., Jiang G.M. (2016). Design and fabrication of auxetic warp-knitted structures with a rotational hexagonal loop. Text. Res. J..

[27] Foroughi J., Spinks G.M., Aziz S., Mirabedini A., Jeiranikhameneh A., Wallace G.G., Kozlov M.E., Baughman R.H. (2016). Knitted carbon-nanotube-sheath/spandex-core elastomeric yarns for artificial muscles and strain sensing. ACS Nano.

[28] Zhang H., Dias T.K. (2016). Electromechanical properties of knitted fabric integrated with laser engraved carbon-loaded fiber. J. Text. Inst..

[29] Tokarska M., Orpel M. (2019). Study of anisotropic electrical resistance of knitted fabrics. Text. Res. J..

[30] Cai G., Yang M., Pan J., Cheng D., Xia Z., Wang X., Tang B. (2018). Large-scale production of highly stretchable cnt/cotton/spandex composite yarn for wearable applications. ACS Appl. Mater. Interfaces.

[31] Xu L., Liu Z., Zhai H., Chen X., Sun R., Lyu S., Fan Y., Yi Y., Chen Z., Jin L. (2020). Moisture-resilient graphene-dyed wool fabric for strain sensing. ACS Appl. Mater. Interfaces.

[32] Li Y.T., Miao X.H., Raji R.K. (2019). Flexible knitted sensing device for identifying knee joint motion patterns. Smart Mater. Struct..

[33] Liu X., Su G., Guo Q., Lu C., Zhou T., Zhou C., Zhang X. (2018). Hierarchically structured self-healing sensors with tunable positive/negative piezoresistivity. Adv. Funct. Mater..

[34] Ryu H., Park S., Park J.J., Bae J. (2018). A knitted glove sensing system with compression strain for finger movements. Smart Mater. Struct..

[35] Hao D.D., Xu B., Cai Z.S. (2018). Polypyrrole coated knitted fabric for robust wearable sensor and heater. J. Mater. Sci. Mater. Electron..

[36] Wang L., Zhang M., Yang B., Tan J., Ding X. (2020). Highly compressible, thermally stable, light-weight, and robust aramid nanofibers/ti3alc2 mxene composite aerogel for sensitive pressure sensor. ACS Nano.

[37] Pan C., Wang J., Ji X., Liu L. (2020). Stretchable, compressible, self-healable carbon nanotube mechanically enhanced composite hydrogels with high strain sensitivity. J. Mater. Chem. C.

[38] Bi L., Yang Z., Chen L., Wu Z., Ye C. (2020). Compressible agnws/ti3c2tx mxene aerogel-based highly sensitive piezoresistive pressure sensor as versatile electronic skins. J. Mater. Chem. A.

[39] Wang J.-J., Zhang Q., Ji X.-X., Liu L.-B. (2020). Highly stretchable, compressible, adhesive, conductive self-healing composite hydrogels with sensor capacity. Chin. J. Polym. Sci..

[40] Song Y., Chen H., Su Z., Chen X., Miao L., Zhang J., Cheng X., Zhang H. (2017). Highly compressible integrated supercapacitor-piezoresistance-sensor system with cnt-pdms sponge for health monitoring. Small.

[41] Jiang X., Ren Z., Fu Y., Liu Y., Zou R., Ji G., Ning H., Li Y., Wen J., Qi H.J. (2019). Highly compressible and sensitive pressure sensor under large strain based on 3d porous reduced graphene oxide fiber fabrics in wide compression strains. ACS Appl. Mater. Interfaces.

[42] Kurbak A. (2017). Geometrical models for weft-knitted spacer fabrics. Text. Res. J..

[43] Souri H., Bhattacharyya D. (2018). Wearable strain sensors based on electrically conductive natural fiber yarns. Mater. Des..

[44] Zhao K., Niu W.B., Zhang S.F. (2020). Highly stretchable, breathable and negative resistance variation textile strain sensor with excellent mechanical stability for wearable electronics. J. Mater. Sci..

